# Tri-Band Vis–NIR Spectroscopy with Color Residual-Variance Gated Attention Fusion for Rapid Assessment of Hongmeiren (*Citrus reticulata*) Soluble Solids Content

**DOI:** 10.3390/foods15173166

**Published:** 2026-09-07

**Authors:** Anan Tao, Longfei Ye, Chaoxu Yu, Liuye Cao, Tiantian Pan, Fei Liu

**Affiliations:** 1 Green Development Center, Bureau of Agriculture and Rural Affairs of Xiangshan County, Ningbo 315799, China; taoanan1984@163.com (A.T.); yu6971@139.com (C.Y.); 2 Zhejiang Key Laboratory of Agricultural Remote Sensing and Information Technology, College of Biosystems Engineering and Food Science, Zhejiang University, 866 Yuhangtang Road, Hangzhou 310058, China; lfye@zju.edu.cn (L.Y.); 12313070@zju.edu.cn (L.C.); ttpan@zju.edu.cn (T.P.); 3 Key Laboratory of Spectroscopy Sensing, Ministry of Agriculture and Rural Affairs, Hangzhou 310058, China

**Keywords:** citrus, soluble solids content detection, Vis–NIR spectroscopy, data fusion, deep learning

## Abstract

Rapid assessment of internal fruit quality is essential for fruit grading, postharvest management, and consumer-oriented quality evaluation. Among the quality attributes, soluble solids content (SSC) is a key indicator of citrus sweetness and maturity. Visible and near-infrared (Vis–NIR) spectroscopy provides an effective approach for rapid SSC detection in fruit. However, most existing studies rely on single full-spectrum models or simple band stacking strategies, which limits their ability to fully exploit complementary information among different spectral sub-bands. To address this limitation, a color residual-variance gated attention fusion network (CR-VGAFNet) is proposed for efficient SSC assessment in Hongmeiren. On the independent prediction set, CR-VGAFNet achieved a prediction correlation coefficient (R_P_) of 0.7744, a root mean square error of prediction (RMSE_P_) of 0.6530 °Brix, and a mean absolute percentage error of prediction (MAPE_P_) of 4.83%. These findings suggest the potential of the framework for multi-band spectral fusion. This study provides a new technical perspective for multi-band spectral fusion and rapid fruit quality assessment.

## 1. Introduction

Hongmeiren (*Citrus reticulata*) is a premium fresh citrus cultivar with high market value. It has attracted considerable market attention because of its tender and juicy pulp, rich flavor, attractive peel color, and high sugar content. In particular, its high sugar content is a major contributor to its desirable flavor and commercial value, making Hongmeiren a representative high-end cultivar in the specialty citrus industry [1]. With the increasing commercialization of Hongmeiren, rapid and accurate assessment of its internal quality is of great importance for fruit grading, postharvest circulation, and consumer experience [2]. Soluble solids content (SSC) is a key indicator of fruit sweetness, maturity, and commercial value, and is therefore an important parameter for evaluating the internal quality of Hongmeiren [3]. SSC measurement usually relies on refractometric or chemical analysis after juice extraction, which is destructive, time-consuming, and difficult to apply to large batches of fruit. These limitations make conventional methods unsuitable for the practical demands of efficient postharvest sorting, storage management, and online quality evaluation [4]. Therefore, developing an efficient method for Hongmeiren SSC detection is essential for improving fruit grading efficiency and quality control.

To enable rapid and nondestructive assessment of internal fruit quality, various techniques have been explored, including hyperspectral imaging (HSI), near-infrared spectroscopy (NIR), visible and near-infrared spectroscopy (Vis–NIR), and laser-induced breakdown spectroscopy (LIBS) [5]. HSI can simultaneously acquire spatial and spectral information, but its high equipment cost, high data dimensionality, and complex modeling and online deployment limit its practical application [6]. LIBS provides high sensitivity for elemental analysis, but existing studies have only achieved indirect semi-quantitative SSC detection through the elemental analysis of citrus leaves, and its application in online fruit sorting is limited by matrix effects and instrument maintenance requirements [7]. Although NIR is convenient for spectral acquisition, it may overlook visible-region information associated with peel color, maturity, and pigment changes [8]. In contrast, Vis–NIR point spectroscopy combines a relatively simple instrumental structure, rapid acquisition, and low cost, making it suitable for large-scale, low-cost, and high-throughput postharvest detection scenarios [9,10]. Therefore, further improving the prediction accuracy and stability of Vis–NIR spectroscopy for Hongmeiren SSC remains an important research issue.

From the perspective of spectral response, different Vis–NIR wavelength regions exhibit different sensitivities to fruit appearance and internal quality attributes, thereby providing multi-source information for Hongmeiren SSC prediction [11]. However, existing studies on fruit SSC modeling have mainly focused on preprocessing methods, feature selection, and often use a single spectral band as the model input [12]. Different Vis–NIR bands may contain complementary quality-related information, and their prediction reliability may vary among samples. Therefore, rational use of different spectral bands and full exploitation of their complementarity and uncertainty during fusion are critical for improving Hongmeiren SSC prediction performance.

In a multi-band modeling framework, the way in which outputs from different band models are fused directly determines the final detection performance. Feature-level fusion methods concatenate features from different bands or auxiliary variables before building a unified regression model, but they are susceptible to high-dimensional redundancy, feature correlation, and overfitting under limited sample sizes. Decision-level fusion methods usually integrate base-model predictions through simple averaging, calibration error-based fixed weighting, or linear stacking, but their weights are typically fixed across all samples and cannot dynamically adjust band contributions according to sample-specific prediction uncertainty [13,14]. In addition, color features are closely related to fruit maturity and external quality and may provide auxiliary information for SSC prediction [15]. However, color features may overlap with spectral principal components, and different color indices may show inconsistent correlation directions with SSC, so direct feature concatenation can introduce interference rather than stable improvement. To address these limitations, a color residual variance-gated attention fusion network (CR-VGAFNet) is proposed for rapid SSC assessment in Hongmeiren using tri-band Vis–NIR spectroscopy. The proposed method uses the frozen Gaussian process regression (GPR) predictive means and variances from each band for variance conditioned, sample adaptive fusion and learns a residual correction from direction aligned color features.

The objectives of this study are threefold. (1) The independent predictive ability of different Vis–NIR bands under different regression models is systematically compared to evaluate their effectiveness and complementarity for Hongmeiren SSC detection. (2) Feature-level fusion, fixed-weight decision-level fusion, and linear stacking are compared to identify the performance bottlenecks of different fusion strategies in multi-band spectral modeling. (3) CR-VGAFNet is developed by integrating a variance-gated attention mechanism with a sign-aligned color residual module to achieve adaptive weighted fusion of multi-band spectral information and color-feature-assisted correction, providing an effective method for rapid SSC detection in Hongmeiren.

## 2. Materials and Methods

### 2.1. Sample Preparation

The primary dataset comprised 360 Hongmeiren samples harvested in early January 2026 from orchards in seven production areas of Xiangshan County, Ningbo City, Zhejiang Province, China: Dandong Subdistrict (*n* = 60), Daxu Town (*n* = 60), Qiangtou Town (*n* = 55), Tuci Town (*n* = 55), Dingtang Town (*n* = 63), Xinqiao Town (*n* = 53), and Xiaotang Township (*n* = 14). In addition, six samples were collected from an independent eighth production site. The complete sample flow is summarized in Table S1. All samples were shipped by express courier immediately after harvest and were delivered to the laboratory within 48 h. Spectral acquisition and reference SSC measurements were initiated after arrival and organized as a sample-wise sequential workflow. Specifically, the Vis–NIR spectrum of each fruit was acquired first, followed immediately by SSC measurement of the same fruit. This procedure minimized the holding interval between spectral acquisition and reference measurement and ensured that the spectral and physicochemical data corresponded to the same sample condition. All spectral acquisition and physicochemical measurements were conducted during the same period. To improve sample representativeness and cover the variation encountered in commercial fruit grading, high-quality and ordinary fruits were selected in appropriate proportions. High-quality fruits were defined as those with intact appearance, uniform coloration, relatively high maturity, and good commercial quality, whereas ordinary fruits had relatively lower appearance uniformity or commercial grade but showed no obvious disease, insect damage, or mechanical injury. Fruits of large, medium, and small size classes were also included to account for variation in fruit size. After harvest, all samples were directly transported by the cooperative producer to Zhejiang University for subsequent spectral acquisition and physicochemical measurement. This sampling design covered differences in production area, quality grade, and fruit size, providing a basis for developing a rapid Hongmeiren SSC detection model.

### 2.2. Vis–NIR and SSC Measurement

Vis–NIR spectra of the Hongmeiren samples were acquired using a three-channel field spectrometer (OFS2500, Oceanhood, Shanghai, China) equipped with a reflectance probe (OFS2500-45/0, Oceanhood, Shanghai, China) under constant low-light laboratory conditions at 25 °C to minimize interference from ambient stray light [16]. The probe incorporated an integrated tungsten lamp and employed a fixed 0° illumination/45° collection geometry to ensure consistent illumination and spectral acquisition conditions across all samples. The three spectral channels were stitched at 950 and 1637 nm, and their integration times were set to 600 μs, 2500 μs, and 17 ms, respectively. Before spectral acquisition, the spectrometer was powered on, the optical fiber path was connected, and the acquisition parameters were configured using the host software. The spectrometer was calibrated against a white-reference panel before spectral acquisition, and the calibration was repeated every 15 min throughout the measurement process. The probe window was then placed in close contact with the peel to prevent gaps at the measurement interface. Spectra were collected at three evenly distributed positions around the equatorial region of each fruit to reduce the influence of tissue heterogeneity. Ten consecutive scans were performed at each position and averaged to obtain a representative spectrum for that position, after which the spectra from the three positions were further averaged to obtain the raw spectrum of each sample. The spectrometer covered the wavelength range of 350–2500 nm, with spectral resolutions of 2.5 nm over 350–950 nm and 8.0 nm over 950–2500 nm. Considering the relatively low signal-to-noise ratios at the edges of the detector range, the 500–2400 nm region was retained for modeling, thereby preserving the effective spectral information from all three channels while excluding the relatively noisy edge bands.

**Figure 1:**
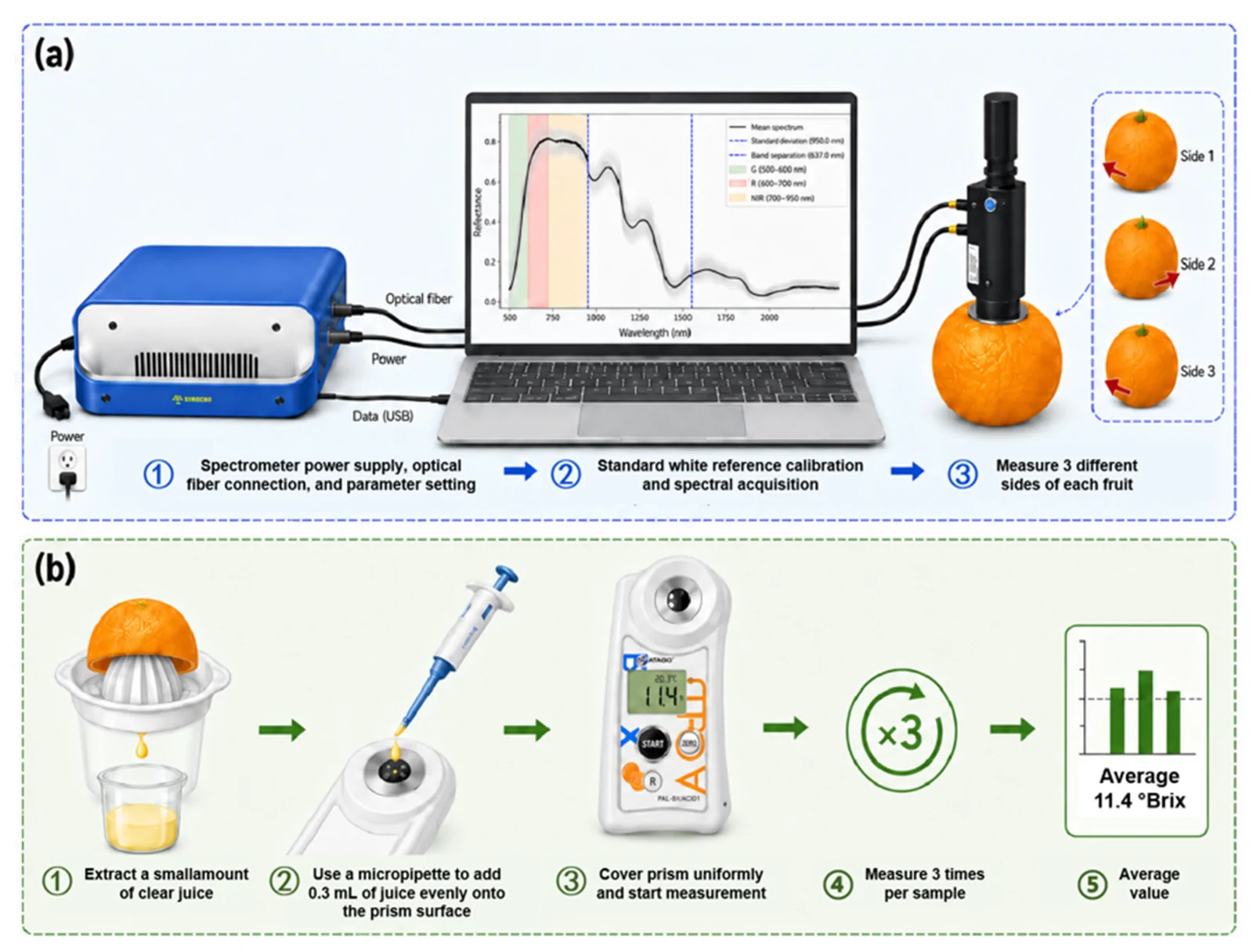
(**a**) Vis–NIR spectral acquisition and (**b**) SSC measurement procedure for the Hongmeiren samples. The illustrative elements were created with the assistance of GPT-5.5 (OpenAI, San Francisco, CA) using the original experimental photographs as visual references. AI assistance was limited to schematic rendering and did not alter the experimental procedures, samples, measurements, or data.

After spectral acquisition, physicochemical measurement of SSC was performed on the same batch of Hongmeiren samples. The pulp of each sample was squeezed and filtered to remove pulp fibers, and the clarified juice was collected for analysis. A 0.3 mL aliquot of juice was uniformly pipetted onto the prism surface of a sugar-acid meter (PAL-BX/ACID 1, ATAGO, Tokyo, Japan), and the detection program was started to record the SSC value of each sample, expressed in °Brix. To reduce random errors caused by manual operation, each juice sample was measured three consecutive times, and the arithmetic mean was used as the final reference SSC value. The measurement procedure is shown in Figure 1b. For the primary modeling dataset, Vis–NIR spectra and corresponding SSC reference values were obtained from 360 samples; six additional samples were analyzed separately for the preliminary independent orchard evaluation.

### 2.3. Data Analysis

#### 2.3.1. Color Residual-Variance Gated Attention Fusion Network

**Figure 2:**
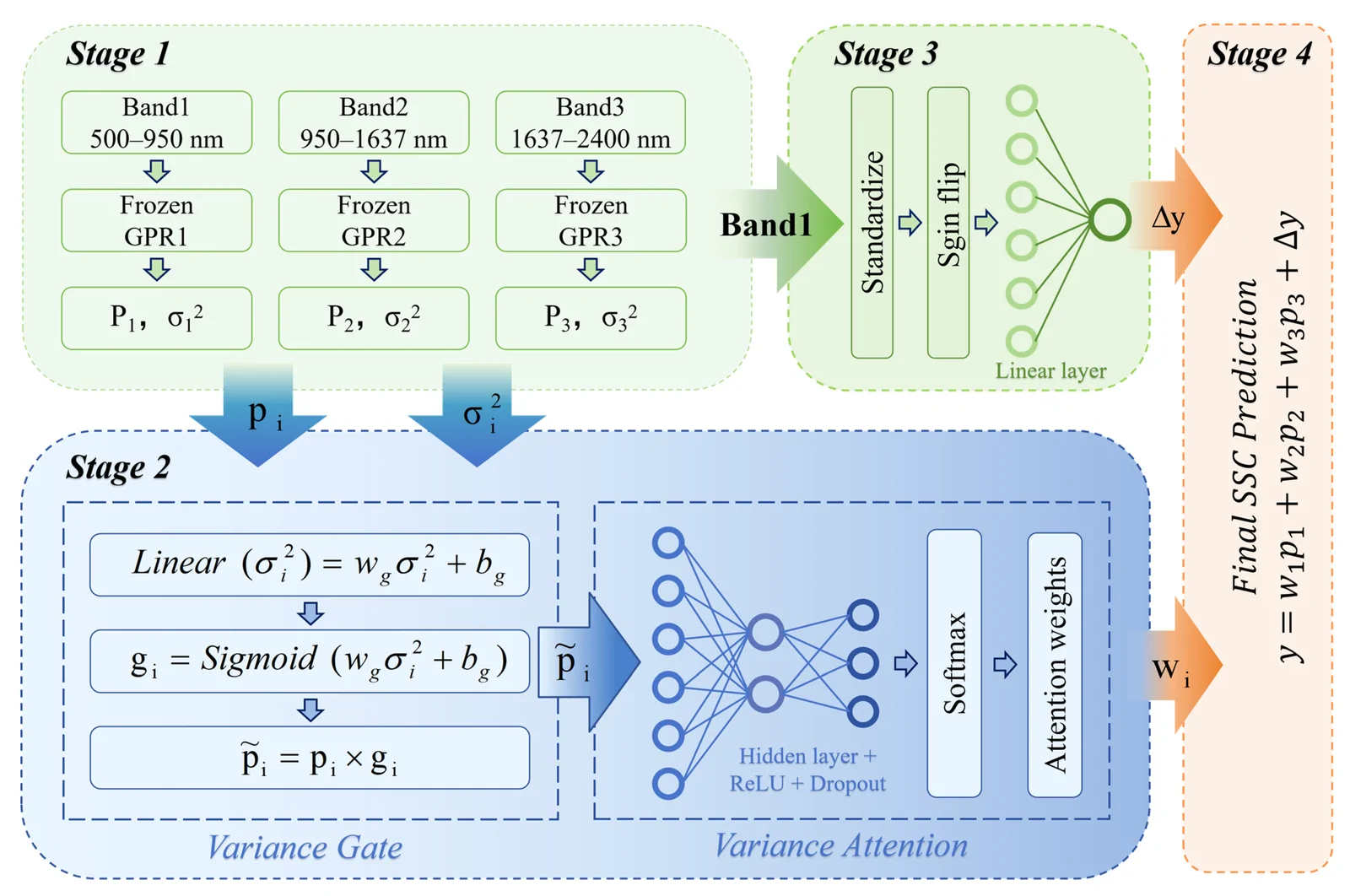
The framework of the CR-VGAFNet. Stage 1: Tri-Band spectral feature extraction; Stage 2: Variance gated attention fusion; Stage 3: Sign-aligned color residual correction; Stage 4: Final SSC prediction.

CR-VGAFNet uses three independent frozen GPR models as preliminary spectral feature extractors, which output the predicted mean and variance for each spectral band. Six color features extracted from Band1 are introduced as auxiliary inputs. The network consists of a variance-gated attention fusion (VGAF) module and a color residual (CR) module, and its overall architecture is shown in Figure 2.

In the VGAF module, a learnable one-dimensional gating transformation is first applied to the variance of each band. The Sigmoid function maps each variance to a gating coefficient between zero and one, which is then multiplied by the corresponding band prediction to generate a variance-modulated prediction. The gating transformation is learned jointly with the attention network. The three modulated predictions and the three original variances are then concatenated into a six-dimensional vector and fed into the attention network. The attention network consists of a hidden layer with two neurons, a rectified linear unit (ReLU) activation function, and a Dropout layer, followed by Softmax normalization to output dynamic fusion weights for the three bands. These weights are used to compute a weighted sum of the original predictions, yielding the attention-fused value. The color residual module consists of an unconstrained linear layer that takes the prior-informed sign-normalized six-dimensional color-feature vector as input and outputs a scalar residual correction. The weights and bias of this linear layer are initialized to zero at the beginning of training. The final network output is obtained by summing the attention-fused value and the color residual value.

Before fusion-model training, the three GPR predictive means, three predictive variances, and six sign-aligned color features were standardized feature-wise. For each model fit, the standardization parameters were estimated exclusively from the corresponding training subset and then applied unchanged to the validation and prediction samples. During training, the mean squared error loss function was used, and adaptive moment estimation (Adam) was adopted as the optimizer with an initial learning rate of 0.001 and a weight decay coefficient of 0.2. The learning rate was automatically reduced using an adaptive decay strategy when the validation loss stopped decreasing. An early stopping strategy was also applied, in which training was terminated and the best weights were restored if the validation loss did not improve for 30 consecutive epochs. For each fusion-network fit, the samples available for second-level training, represented by the first-level OOF GPR outputs, were randomly divided into an optimization subset (90%) and an internal validation subset (10%) using random seed 42.

#### 2.3.2. Quantitative Models, Optimization, and Metrics

Before model construction, the dataset was sorted by SSC and divided into a calibration set of 268 samples and an independent prediction set of 92 samples using interval sampling, ensuring a balanced SSC distribution in both subsets. A variety of preprocessing methods were then applied to the raw spectra, including Savitzky–Golay smoothing (SG), standard normal variate correction (SNV), multiplicative scatter correction (MSC), first derivative (1stDeriv), and combinations of SG with SNV, MSC, and first derivative, namely SG + SNV, SG + MSC, and SG + 1stDeriv [17,18]. This comprehensive preprocessing search space was designed to reduce baseline drift, scattering effects, and random noise while preserving spectral absorption features. Principal component analysis (PCA) was subsequently used to compress the preprocessed spectra and extract the major sources of variation as model inputs, thereby further reducing collinearity and redundant information [19].

To establish quantitative relationships between Vis–NIR spectra and Hongmeiren SSC and to evaluate the effectiveness of CR-VGAFNet, four additional regression models were employed: partial least squares regression (PLSR), support vector regression (SVR), GPR, and one-dimensional convolutional neural network (1D-CNN) [20,21,22,23]. These models cover different paradigms, including linear modeling, nonlinear kernel methods, Bayesian nonparametric modeling, and deep learning, enabling a comprehensive evaluation of the predictive ability of each Vis–NIR spectral band for Hongmeiren SSC. PLSR is a multivariate analysis method particularly suitable for high-dimensional and highly collinear spectral data. It projects independent and dependent variables into a low-dimensional latent-variable space and identifies linear combinations that maximize their covariance, thereby avoiding the instability of conventional regression under collinearity [24]. SVR is based on support vector machine theory and uses kernel functions to map low-dimensional spectral data into a high-dimensional space to capture complex nonlinear relationships. Its ε-insensitive loss function allows a predefined error tolerance and relies only on support vectors for fitting, making it suitable for high-dimensional nonlinear data [25]. GPR is a Bayesian nonparametric method that introduces a prior distribution over functions and updates it using observed data [26]. It provides both point predictions and predictive variances for uncertainty estimation, which serve as key inputs to CR-VGAFNet. 1D-CNN applies one-dimensional convolutional filters along the spectral dimension and can automatically learn hierarchical features. Although it has shown potential in chemometrics, strict regularization is required under limited sample sizes to avoid overfitting [27].

For PLSR, the number of latent variables was optimized. For SVR, the penalty parameter C, kernel coefficient γ, and ε-tube width were tuned under both linear and radial basis function kernels. For GPR, combinations of different kernels, including radial basis function, Matern, rational quadratic, and white-noise kernels, were searched. For 1D-CNN, the number of convolutional kernels, number of fully connected units, and Dropout ratio were optimized. All models were developed using the same calibration and independent prediction sets, identical fivefold partitions, and a fixed random seed, with all model selection confined to the calibration set. For models requiring second-level learning or GPR-derived inputs, out-of-fold (OOF) GPR predictions were used. Within each outer fold, spectral preprocessing, PCA, and GPR hyperparameters were optimized using only the corresponding outer-training subset, and the fitted GPRs generated predictive means and variances for the held-out samples without being updated during second-level model training. The preprocessing methods and model hyperparameters for all models, together with the fold-specific preprocessing, PCA, and GPR configurations used in the OOF procedure, are provided in Supplementary Tables S2–S7. The independent prediction set was kept completely separate from preprocessing selection, hyperparameter optimization, and model training, and was used only for final performance evaluation. Model performance was evaluated using three commonly used metrics: correlation coefficient (R), RMSE, and mean absolute percentage error (MAPE) [28]. R reflects the linear correlation between the predicted and reference values, RMSE quantifies the overall prediction error, and MAPE evaluates the prediction accuracy in terms of relative error. Higher R values and lower RMSE and MAPE values indicate better predictive performance.

## 3. Results and Discussion

### 3.1. Analysis of Vis-NIR Spectral Responses

**Figure 3:**
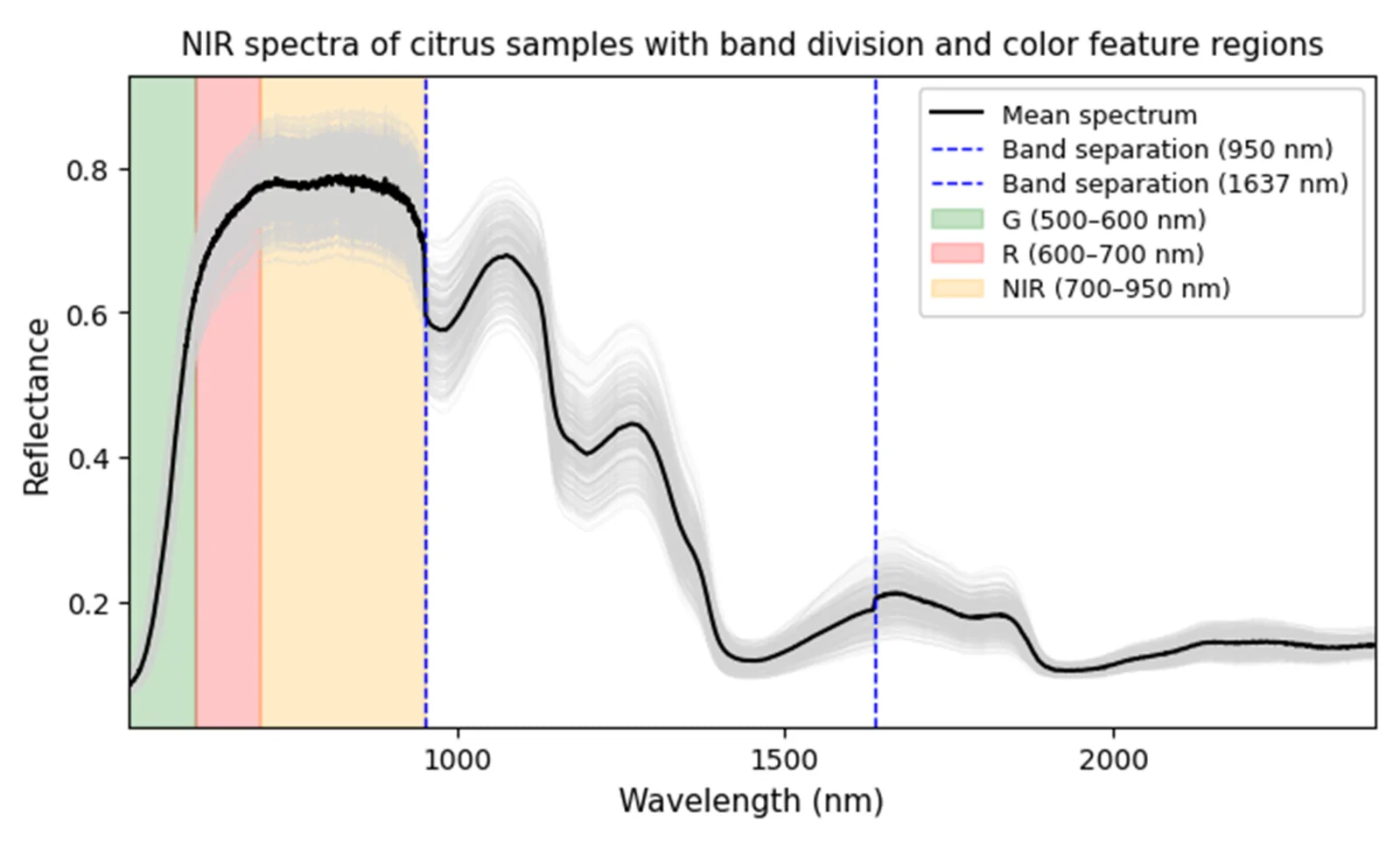
Vis–NIR spectra of Hongmeiren samples with band division and color feature extraction regions.

Vis–NIR spectra exhibit distinct responses to the internal chemical composition of Hongmeiren across different wavelength regions, which in turn affects model performance in SSC prediction [3]. Figure 3 shows the mean spectra of all samples, the extraction regions of the color features G, R, and NIR, and the boundaries of the three spectral bands. Band1 (<950 nm) covers the visible and short-wave near-infrared regions. This band contains color-related information associated with fruit appearance and can also reflect the absorption characteristics of some pigments in the peel [29]. Band2 (950–1637 nm) corresponds to a typical near-infrared region and contains overtone and combination absorption information related to hydrogen-containing groups, which is closely associated with molecular vibrations of soluble components such as sugars and organic acids [2,3]. Band3 (>1637 nm) mainly represents the long-wave near-infrared region. Although this region usually has a relatively low signal-to-noise ratio because of stronger absorption by water and sugars, it still contains useful information that is not available in shorter wavelengths [11]. The green (G, 500–600 nm), red (R, 600–700 nm), and near-infrared (NIR, 700–950 nm) regions are further marked within Band1 using semi-transparent colored rectangles. The mean reflectance of each corresponding region was used to describe the spectral response of peel from the visible to short-wave near-infrared range. Based on these three basic color features, three ratio indices were further constructed: the green-to-red ratio (G/R), the near-infrared-to-red ratio (NIR/R), and the normalized difference vegetation index (NDVI). Unlike conventional colorimetric measurements, these variables and indices were calculated directly from the Vis–NIR reflectance spectra and provide a low-dimensional description of peel-related spectral characteristics. In this study, the three bands were modeled independently, and color features were fused in different ways. This strategy preserves information from different physicochemical sources, provides diverse feature sources for subsequent fusion, and avoids the averaging effect that may occur when a single model is applied to the full spectral range.

### 3.2. Performance Investigation of Single-Band-Based Models

To preliminarily investigate the predictive performance of different bands under different types of machine learning models, four models were constructed for each band: the linear model PLSR, the nonlinear model SVR, the probabilistic model GPR, and the deep learning model 1D-CNN for one-dimensional spectra. As shown in Table 1, GPR achieved the best prediction performance in all three bands, with R_P_ values of 0.6819, 0.7003, and 0.6990, respectively, which were higher than those of PLSR and SVR. The corresponding RMSE_P_ values were 0.7637, 0.7650, and 0.7427 °Brix, and the MAPE_P_ values were also the lowest among all models. PLSR and SVR generally showed a similar performance but were clearly inferior to GPR. In contrast, 1D-CNN produced the poorest results, especially in Band1 and Band3, where RMSE_P_ exceeded 1 °Brix, indicating very limited generalization ability.

The advantage of GPR can be attributed to its nonparametric Bayesian inference framework. Through prior assumptions over the function space and kernel selection, GPR can flexibly capture the complex nonlinear relationship between spectral features and SSC. Meanwhile, its built-in marginal likelihood can automatically balance model complexity, thereby effectively suppressing overfitting. This is particularly important for a small one-dimensional spectral dataset with only 268 calibration samples. In comparison, PLSR, as a linear latent-variable model, has limited ability to fit nonlinear spectral–SSC relationships. Although SVR can extend nonlinear representation through the kernel trick, its performance is highly dependent on sensitive hyperparameter tuning. 1D-CNN has strong feature extraction potential, but under small-sample conditions, excessive parameters can easily lead to a sharp decline in generalization ability. The inferior performance of the 1D-CNN should not be interpreted as evidence that deep learning is generally unsuitable for Vis–NIR regression. Rather, the relative performance of deep and conventional models depends strongly on sample size, cultivar characteristics, spectral acquisition mode, and model complexity. For example, a recent comparative study involving Ponkan and Tianchao citrus found that PLSR achieved the best result for Ponkan, whereas CNN performed relatively better for Tianchao [30]. In addition, the GPR models using three independent and non-overlapping bands achieved comparable prediction performance, indicating that each band independently contains effective information related to SSC. Therefore, how to efficiently integrate the complementary information contained in these three independent bands becomes a key issue. In the following sections, different fusion strategies are compared to identify an optimal fusion scheme that can fully exploit the useful information from each band.

### 3.3. Comparison of Multi-Band and Color Feature Fusion Strategies

To fully exploit the complementary information provided by multiple bands, it is necessary to design an appropriate fusion strategy. Common fusion strategies can be divided into two categories. The first is feature-level fusion, in which features from different sources are concatenated at the feature level and then fed into a single regression model. The second is decision-level fusion, in which the predictions of base models are first obtained and then integrated using fixed rules or learnable methods [14]. This section systematically discusses the performance of these two types of fusion methods and the proposed CR-VGAFNet in Hongmeiren SSC prediction.

#### 3.3.1. Feature-Level Fusion and Directional Consistency Analysis of Color Features

This section aims to determine whether concatenating PCA features from different spectral bands with color features at the feature level can provide additional information gain for Hongmeiren SSC prediction. To this end, six color features were added to the inputs of the three single bands and All Bands, respectively, and the results were compared with those obtained using spectral features alone. Table 2 summarizes the prediction results of the optimal models on the independent prediction set. As shown in Table 2, directly concatenating the spectral features of the three bands and feeding them into a single GPR model increased R_P_ to 0.7444 and reduced RMSE_P_ to 0.6904 °Brix, representing a clear improvement over any single-band GPR model. Among the three single-band GPR models, Band2 achieved the highest R_P_ (0.7003), whereas Band3 yielded the lowest RMSE_P_ (0.7427 °Brix) and MAPE_P_ (5.29%). This indicates that the spectral information from the three bands is complementary, and that simple feature concatenation can effectively integrate multi-band advantages and improve prediction accuracy. However, after further introducing color features, the results changed. For single-band spectra, the contribution of color features was unstable. The performance of Band1 degraded after adding color features, with R_P_ decreasing from 0.6819 to 0.6633. Band2 showed an almost unchanged performance, with R_P_ changing from 0.7003 to 0.7009. Only Band3 showed a relatively clear improvement, with R_P_ increasing from 0.6990 to 0.7247. For the full-band input, R_P_ slightly decreased from 0.7444 to 0.7414 after adding color features, while RMSE_P_ increased from 0.6904 to 0.7167 °Brix, indicating that the expected positive gain was not achieved. The fundamental reason for this phenomenon lies in the source and information attributes of the color features. All six color features were extracted from the original reflectance spectra of Band1 (<950 nm), while Band1 had already been sufficiently compressed using PCA. Therefore, the information contained in the color features overlapped with the original information represented by the Band1 PCA features. Directly concatenating the two types of features instead introduced redundant noise, leading to degraded fusion performance for Band1. In contrast, the spectral ranges of Band2 and Band3 did not overlap with Band1, so there was no direct information redundancy between their PCA features and the color features. The color features could therefore provide some complementary information related to SSC, resulting in maintained or improved performance. However, when all three bands were concatenated, the redundancy between the color features and Band1 diluted the slight gains observed in Band2 and Band3, ultimately resulting in no clear improvement, or even a slight decline, in full-band fusion.

**Figure 4:**
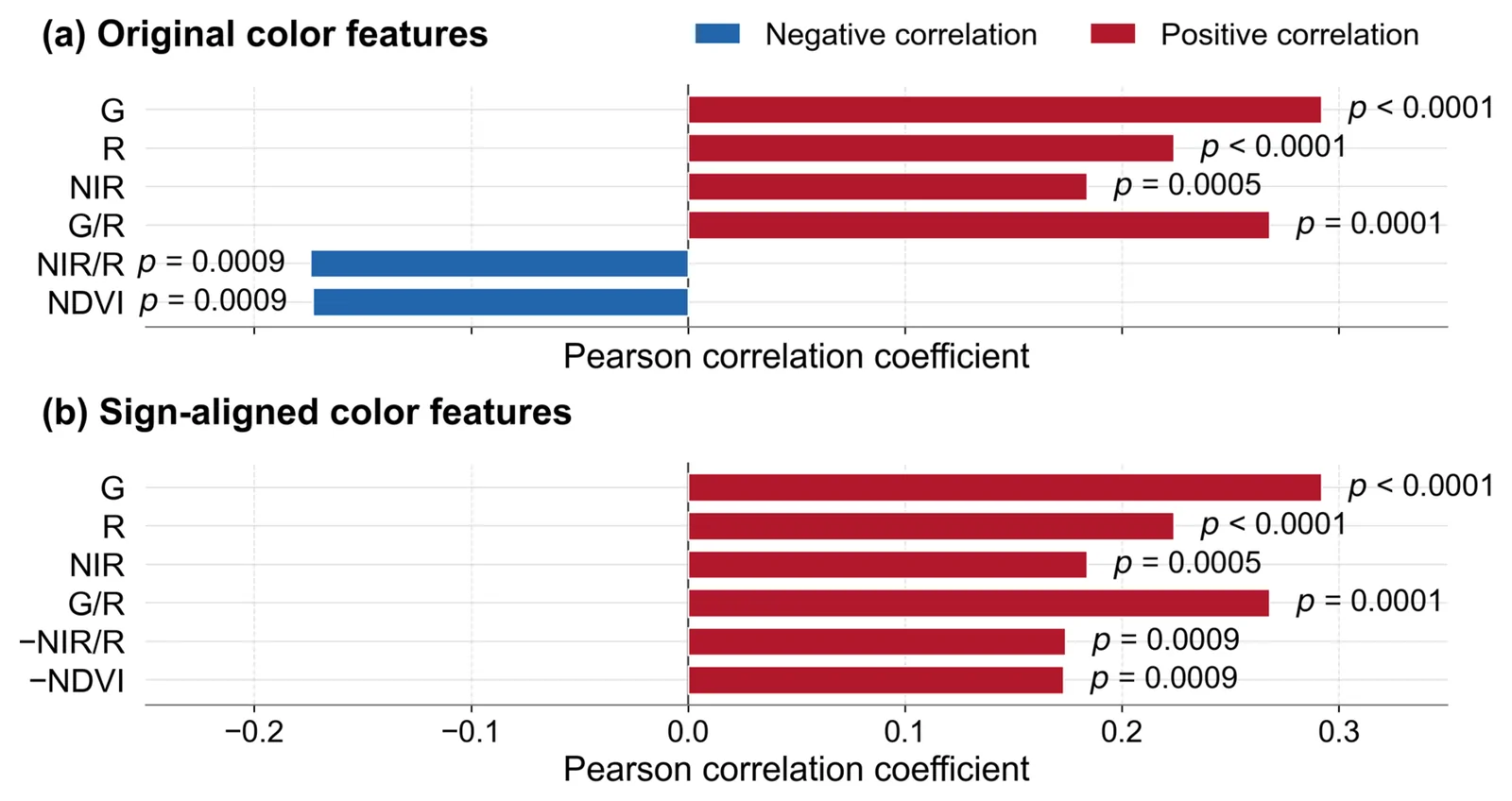
The correlation between color features and Hongmeiren SSC before and after sign flipping: (**a**) original color features and (**b**) direction-normalized color features.

The above results indicate that simple feature-level concatenation can lead to information overlap and redundancy between the color features and Band1, preventing effective use of color information. To further investigate why the direct fusion of color features failed to improve quantitative performance, the linear relationships between the six color features and Hongmeiren SSC were evaluated by calculating the Pearson correlation coefficients and significance levels using all calibration samples. The absolute value of the Pearson correlation coefficient indicates the strength of the linear relationship, its sign indicates the direction of correlation, and the *p*-value indicates statistical significance [31]. The results are presented as a correlation heatmap in Figure 4.

As shown in Figure 4, the G, R, NIR, and G/R were positively correlated with SSC, whereas the NIR/R and NDVI were negatively correlated with SSC. Although the absolute Pearson correlation coefficients between the individual color features and SSC were below 0.3, all correlations were statistically significant (*p* < 0.001), indicating weak but non-random marginal linear associations. If properly used in modeling, they may still provide additional useful information for SSC prediction. However, the different correlation directions prevented the original color features from having a consistent directional interpretation with respect to SSC. Moreover, the results of the feature-level fusion models showed that simply concatenating the color and spectral features did not effectively translate these weak associations into improved prediction performance. This result motivated the use of prior-informed direction normalization in the color residual module of CR-VGAFNet: the signs of the negatively correlated features were reversed based exclusively on the calibration data so that increasing transformed feature values consistently represented an increasing SSC tendency. This preprocessing step facilitated the interpretation of the color features and their corresponding coefficients in the residual layer, after which the color features were used to learn a residual correction to the fused spectral prediction. Therefore, although the three bands describe the same SSC-related property, they do not provide completely redundant predictions. Their differences reflect the heterogeneous responses of different spectral and color representations to sample composition and surface characteristics. This type of complementary information has also motivated recent applications of multi-source and multi-task learning in Vis–NIR fruit-quality analysis, in which jointly exploiting related representations improved the robustness of internal-quality prediction [32]. Accordingly, the observed directional consistency and residual differences among the three bands provide the basis for the subsequent decision-level fusion.

#### 3.3.2. Decision-Level Fusion Strategies and Predictive Performance of CR-VGAFNet

Decision-level fusion directly integrates the predictions of base models from different bands and no longer involves re-concatenation of the original spectral variables. To further distinguish the influence of color features on decision-level fusion, two types of base-model inputs were constructed. One used only the spectral PCA features of each band, whereas the other further concatenated color features with the spectral PCA features of each band. Then, based on the GPR predictions from the three bands, three representative decision-level fusion strategies were compared: direct averaging of predictions (Average), inverse calibration set RMSE-based weighting (RMSE-based weighting), and ridge-regression-based linear stacking (linear stacking). To further evaluate whether GPR predictive variances could improve decision-level fusion, two uncertainty-aware methods were additionally included: variance weighting and linear stacking (predictions + variances). The former calculated sample-specific fusion weights from the inverse predictive variances, whereas the latter used the three GPR predictions and their corresponding variances as inputs to ridge regression. These methods were compared with the proposed CR-VGAFNet, and the results are summarized in Table 3.

As shown in Table 3, when only spectral features were used, Average and RMSE-based weighting achieved R_P_ values of 0.7578, with RMSE_P_ values of 0.7235 °Brix and 0.7233 °Brix, respectively. After adding color features, their R_P_ values slightly decreased to 0.7377 and 0.7380, respectively, but RMSE_P_ and MAPE_P_ did not show stable improvement. This indicates that although color features can provide some error compensation for certain fixed-weight fusion strategies, Average and RMSE-based weighting use global fixed weights and cannot dynamically adjust the contribution of each band according to sample-specific spectral response differences. Therefore, they cannot fully release the benefits of color information. For variance-based weighting, adding color features decreased R_P_ from 0.7648 to 0.7390 and increased RMSE_P_ from 0.7166 to 0.7191 °Brix. For linear stacking (predictions), adding color features decreased R_P_ from 0.7496 to 0.7323 and increased RMSE_P_ from 0.6974 to 0.7048 °Brix. Including predictive variances did not improve linear stacking under either input setting. Its overall prediction performance remained lower than that of the fixed-weight strategies and CR-VGAFNet, and its calibration set fitting accuracy was obviously high, indicating that linear stacking is still prone to overfitting when the sample size is limited and base-model predictions are highly correlated.

In contrast, CR-VGAFNet achieved the best performance on the independent prediction set. Its learned fusion framework jointly uses the band-specific GPR predictions and standardized predictive variances to generate sample-dependent fusion weights, thereby avoiding the limitations of fixed weighting and linear stacking methods. Its advantage mainly comes from variance-conditioned adaptive band fusion. The variance-gated attention mechanism dynamically assigns fusion weights according to differences in prediction among bands for each sample, thereby avoiding the limitation of fixed-weight methods that ignore sample heterogeneity. This design preserves the better role of multi-band spectral prediction while avoiding redundant interference and overfitting risks caused by simple feature concatenation and linear stacking. This interpretation is consistent with recent developments in robust spectral analysis, which emphasize that model performance can be improved by incorporating data structure, complementary information, and knowledge-guided constraints rather than merely increasing network depth [33].

Taken together, the failure of feature-level fusion can mainly be attributed to information overlap between color features and spectral PCA features. Such information redundancy makes it difficult for the model to distinguish the relative importance of different features and extract an independently contributing correction signal from color information. In decision-level fusion, fixed-weight methods are limited because they do not account for sample-specific differences. Linear stacking introduces trainable parameters, but it is prone to overfitting when both the sample size and the number of features are limited, thereby impairing generalization ability. CR-VGAFNet successfully overcomes these limitations through the integration of the variance-gated attention fusion mechanism and color residual correction. The negatively correlated color features were direction-normalized to provide a consistent interpretation with respect to SSC, after which the color residual layer learned a correction to the attention-fused prediction. Finally, through the residual connection, the color correction term acts as a fine adjustment to the attention-fused value. This preserves the dominant role of the attention module while allowing fine compensation when necessary, thereby improving prediction accuracy without markedly increasing the risk of overfitting. Through the combined use of variance-conditioned adaptive fusion and residual correction, CR-VGAFNet achieved the best numerical prediction performance on the independent prediction set, providing an effective end-to-end solution for multi-band near-infrared spectral fusion analysis.

#### 3.3.3. Visual Analysis of Prediction Distributions Under Different Fusion Strategies

**Figure 5:**
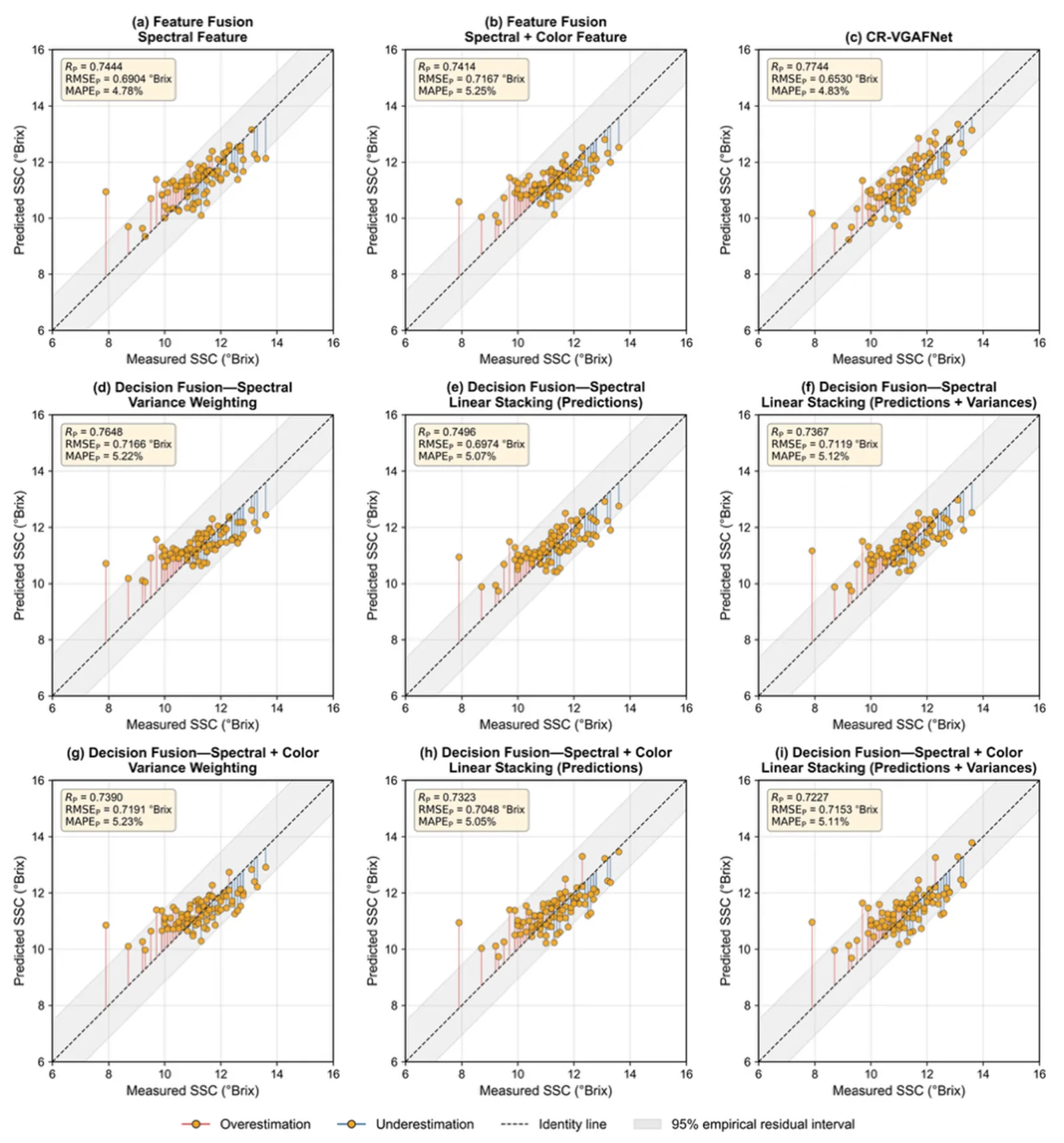
Scatter plots of measured and predicted Hongmeiren SSC under different fusion strategies: (**a**) feature-level fusion using spectral features; (**b**) feature-level fusion using spectral and color features; (**c**) CR-VGAFNet; (**d**) decision-level fusion using spectral features with variance weighting; (**e**) decision-level fusion using spectral features with linear stacking based on predictions; (**f**) decision-level fusion using spectral features with linear stacking based on predictions and predictive variances; (**g**) decision-level fusion using spectral and color features with variance weighting; (**h**) decision-level fusion using spectral and color features with linear stacking based on predictions; and (**i**) decision-level fusion using spectral and color features with linear stacking based on predictions and predictive variances. Vertical segments indicate sample-level residual magnitude and direction (red: overestimation; blue: underestimation); the shaded region represents the 95% empirical residual interval.

To visually compare the prediction performance of different fusion strategies on the independent prediction set, Figure 5 visualizes the agreement between the measured and predicted SSC values for nine fusion strategies. Figure 5a and Figure 5b represents feature-level fusion using spectral features and spectral-plus-color features, respectively, whereas Figure 5c corresponds to CR-VGAFNet. Figure 5d–f shows the decision-level fusion results of variance weighting, linear stacking (predictions), and linear stacking (predictions + variances) using only spectral features, while Figure 5g–i shows the corresponding results after incorporating color features.

Compared with the conventional fusion strategies, CR-VGAFNet produced a more compact scatter distribution around the ideal prediction line, indicating better agreement between the measured and predicted SSC values. This pattern is also reflected by the generally shorter vertical residual segments in Figure 5c, providing more direct sample-level evidence of the reduced prediction errors achieved by CR-VGAFNet. In contrast, feature-level fusion and decision-level fusion showed more dispersed point clouds, with several samples deviating noticeably from the diagonal line. This visual pattern suggests that simple feature concatenation or conventional decision-level integration is less effective in maintaining stable prediction behavior across samples.

It is also worth noting that several low-SSC samples exhibited relatively large deviations in the conventional fusion models, especially when their predicted values were located clearly above the ideal line. The red residual segments further confirm that overestimation was the predominant error direction for these low-SSC samples. In contrast, several samples at the higher end of the SSC range showed blue residual segments, indicating a tendency toward underestimation in some conventional fusion models. These samples may correspond to cases with atypical spectral responses or weaker SSC-related signals, for which direct feature concatenation is less adaptive. CR-VGAFNet alleviated these deviations to some extent by dynamically adjusting the contribution of each band according to sample-specific prediction uncertainty, thereby improving the robustness of prediction for difficult samples.

Overall, CR-VGAFNet maintained a relatively balanced scatter distribution across the 7–14 °Brix range, with no obvious systematic deviation. Both positive and negative residuals were distributed across the SSC range, while their magnitudes were generally smaller than those observed for the conventional fusion strategies. This observation is consistent with the quantitative results in Table 3 and further supports that variance-gated adaptive weighting and color residual correction can improve prediction stability across Hongmeiren samples with different SSC levels.

To examine whether the GPR predictive variance provided a meaningful indication of prediction uncertainty, the 92 samples in the independent prediction set were divided into five quintiles according to the predictive variance of each spectral band. The MAE and RMSE were calculated for each quintile, and the corresponding 95% confidence intervals were estimated using 10,000 bootstrap resamples (Figure S1). For Band1, both MAE and RMSE increased consistently with the predictive-variance quintile, indicating that higher predictive variance was generally associated with larger prediction errors. Band2 exhibited a weaker and non-monotonic relationship, although relatively large errors occurred in the highest-variance quintile. In contrast, Band3 showed no consistent relationship between predictive variance and prediction error. These findings indicate that the uncertainty information provided by GPR predictive variance was band-dependent rather than uniformly reliable across all three bands.

### 3.4. Ablation and Complementary Contribution Analysis of Core CR-VGAFNet Modules

CR-VGAFNet consists of two high-level modules: the variance-gated attention fusion (VGAF) module and the color residual (CR) module. For a more detailed ablation analysis, the VGAF module was further divided into two functional components, namely variance gating and variance attention. Accordingly, as shown in Table 4, six ablation configurations were constructed by selectively enabling or disabling three functional components: variance gating, variance attention, and color residual correction.

Model 1 used only inverse RMSE weighting to fuse the three GPR predictions, achieving R_P_ = 0.7380 and RMSE_P_ = 0.7205 °Brix. The fixed-weight linear ensemble lacked adaptability to sample differences, resulting in limited prediction accuracy. The independent contribution of the CR module was demonstrated by comparing Model 1 and Model 2. Model 2 added only the color residual module to the fixed-weight baseline, increasing R_P_ to 0.7615 and reducing RMSE_P_ to 0.6705 °Brix. This indicates that even when the fusion weights are static, incorporating color information through the residual branch can provide a learned correction to the fixed-fusion prediction. The independent contribution of the VGAF module is demonstrated by comparing Model 1 and Model 3. Model 3 activated the complete VGAF module, in which the learned variance-conditioned gate modulated the band-specific predictions and the attention network dynamically generated fusion weights for each sample. Without using the color residual module, R_P_ increased to 0.7579 and RMSE_P_ decreased to 0.6826 °Brix. Notably, the performance of Model 3 was almost identical to that of Model 2, indicating that either dynamic fusion alone or color residual correction alone can improve the prediction accuracy to a similar level, although their improvement mechanisms are different. The former relies on sample-specific adaptive weighting, whereas the latter relies on prior-guided residual compensation. In addition, the independent effects of gating and attention were further revealed by Model 4 and Model 5. Model 4 removed the variance gating and retained only the attention network and color residual module, achieving R_P_ = 0.7576. Model 5 removed the attention network and retained only variance gating and the color residual module, achieving R_P_ = 0.7574, which was even slightly lower than that of Model 2. This phenomenon indicates that when variance gating and the attention network operate independently, the additional gain is almost negligible. The two components are therefore not simply additive, but complementary contributions. Complete Model 6 activated all three components: variance gate, variance attention, and color residual. It achieved an R_P_ = 0.7744, RMSE_P_ = 0.6530 °Brix, and MAPE_P_ = 4.83%, with a 4.93% improvement in R_P_ over the baseline model. The complete configuration achieved the best numerical performance among the ablation variants, indicating that the modules provided complementary contributions when combined. The underlying mechanism is that the VGAF module provides a fusion starting point closer to the true value for each sample through dynamic attention, reducing the error of the attention-fused value. At this point, the CR module only needs to finely compensate for the remaining small bias, reducing the magnitude of the correction term and thereby lowering the overfitting risk of residual learning under limited sample conditions.

In summary, the ablation experiments reveal the distinct and complementary roles of the VGAF and CR modules in CR-VGAFNet. The VGAF module provides the model with variance-conditioned dynamic fusion ability, enabling each sample to obtain adaptive band weights. The color residual module incorporates complementary information from the color features and provides a learned correction in residual form, whereas direction normalization is used to maintain a consistent interpretation of the feature directions. Each module contributed to prediction performance, while their combination achieved the best numerical result among the evaluated configurations. These results fully verify the rationality and effectiveness of the CR-VGAFNet architecture.

### 3.5. Additional Evaluation, Limitations, and Future Perspectives

To further evaluate the effectiveness and computational efficiency of the proposed variance-gated attention mechanism, CR-VGAFNet was compared with a CBAM-style branch attention model and a multi-head self-attention (MHA) fusion model. CBAM was selected because its number of trainable parameters was comparable to that of CR-VGAFNet, whereas MHA represented a more complex attention architecture with a substantially larger parameter count. For a fair comparison, the three models used identical OOF-generated GPR predictive means and variances, color features, color-residual branches, data partitions, and training settings; only the attention-fusion module was replaced. Because the multiple-random-seed analysis was conducted only for CR-VGAFNet, the results in Table S8 were obtained using the common fixed random seed of 42.

As shown in Table S8, CR-VGAFNet achieved the highest R_P_ (0.7744) and the lowest RMSE_P_ (0.6530 °Brix) among the three models. The CBAM model had a comparable number of trainable parameters (31 versus 32) but produced a lower R_P_ (0.7571), a higher RMSE_P_ (0.6768 °Brix), and a slightly higher MAPE_P_ (4.84% versus 4.83%). The MHA model achieved a slightly lower MAPE_P_ (4.79%) than CR-VGAFNet, but its R_P_ was lower (0.7614) and its RMSE_P_ was higher (0.6705 °Brix). Moreover, MHA required 104 trainable parameters, approximately 3.25 times that of CR-VGAFNet, and its reported prediction time was approximately 11.9% longer. Therefore, although MHA showed a small advantage in MAPE_P_, CR-VGAFNet provided a more favorable overall balance among correlation, absolute prediction error, model complexity, and computational efficiency. These results suggest that increasing attention complexity did not consistently improve prediction performance under the present experimental conditions.

Following the ablation comparison in Table 4, the run-to-run variability of CR-VGAFNet was further examined because its optimization involves stochastic components. Ten independent training runs were conducted using random seeds of 2, 12, 22, 32, 42, 52, 62, 72, 82, and 92, while the calibration/prediction partition, fivefold partitioning, OOF GPR inputs, and early-stopping subset were kept unchanged. Across the 10 runs, CR-VGAFNet obtained an R_P_ of 0.7713 ± 0.0043, an RMSE_P_ of 0.6627 ± 0.0061 °Brix, and a MAPE_P_ of 4.84 ± 0.02% (mean ± SD) on the independent prediction set. The relatively small standard deviations indicate limited sensitivity to stochastic network initialization and training. Using the second best variance-based weighting in Table 3 as the reference, a paired sample-wise comparison was subsequently performed on the same 92 independent prediction samples. Based on the seed-averaged CR-VGAFNet predictions, the RMSE difference was 0.0538 °Brix, corresponding to a numerical reduction of 7.51%. The 95% confidence interval obtained from 10,000 paired bootstrap resamples ranged from −0.1271 to 0.0285 °Brix. The paired absolute-error effect size was small (Cohen’s d_z_ = −0.089). These results demonstrate the repeatability of CR-VGAFNet and indicate a numerical reduction in prediction error relative to RMSE-based weighting, although the statistical evidence for this improvement remains inconclusive under the present sample size.

Several limitations should be considered when interpreting these results. First, the paired analysis was based on only 92 independent prediction samples, which limited the statistical power and contributed to the relatively wide confidence interval. Second, the error reduction was heterogeneous across samples and was partly attributable to improved predictions for a subset of samples with relatively large baseline errors, rather than to a uniform improvement across the entire prediction set. Finally, fixing the data partition, OOF inputs, and early-stopping subset allowed the variability associated with stochastic neural-network training to be isolated, but did not quantify the variability arising from alternative data partitions or external sample sources. Further validation using larger and externally collected datasets is therefore required to determine whether the observed numerical improvement is reproducible across different sample populations and experimental conditions.

Despite the observed improvement, several limitations should be acknowledged. First, all samples were obtained during one harvest period and were measured under controlled laboratory conditions using the same instrument. Although samples from seven production areas were included, the present results do not yet demonstrate robustness across cultivars, seasons, instruments, or substantially different environmental conditions. To further examine model performance for samples from an additional production area, CR-VGAFNet was applied to six representative samples from Xiaotang Township, which covered the A/B and L/M/S categories and were obtained from an independent orchard not used in the original dataset. The sample-level prediction results are presented in Table S9. The model achieved an RMSE of 0.808 °Brix and a MAPE of 5.14%. Nevertheless, owing to the limited number of samples, these results should be regarded as a preliminary production-area evaluation rather than conclusive evidence of broad geographical generalizability. Second, the predictive variances used by CR-VGAFNet were generated by the fitted GPR models and are therefore dependent on the selected kernels, preprocessing procedures, and training distribution; they should be interpreted as model-based uncertainty indicators rather than direct measurements of prediction error. Third, the color variables were derived from the same spectral acquisition rather than from an independent imaging sensor. Future studies should therefore compare CR-VGAFNet with recent self-attention, cross-attention, and multimodal fusion architectures; evaluate the proposed method using multi-season and multi-cultivar datasets collected from external orchards and different instruments; further calibrate its uncertainty estimates; and integrate independent RGB or hyperspectral information. Particular attention should also be given to model lightweighting, inference efficiency, and robustness under varying conveyor speeds, fruit orientations, and ambient conditions, thereby facilitating real-time SSC detection and practical deployment on industrial fruit-grading and processing lines.

## 4. Conclusions

This study proposed CR-VGAFNet for rapid SSC detection in Hongmeiren using tri-band Vis–NIR spectral fusion. The framework integrates frozen GPR predictors, variance-guided VGAF, and CR correction to achieve adaptive band fusion and refined prediction. CR-VGAFNet achieved an R_P_ = 0.7744, RMSE_P_ = 0.6530 °Brix, and MAPE_P_ = 4.83% on the independent prediction set, outperforming conventional fusion strategies. Ablation results indicated that VGAF and CR provided complementary contributions through variance-conditioned fusion and color residual correction, respectively. These results indicate that CR-VGAFNet provides an effective uncertainty-informed modeling framework for multi-band Vis–NIR fusion and the rapid assessment of internal fruit quality.

## Figures and Tables

**Table 1:** Prediction performance of different single-band Vis–NIR models for Hongmeiren SSC.

Model	Band1 (<950 nm)			Band2 (950–1637 nm)			Band3 (>1637 nm)		
	R_P_	RMSE_P_ (°Brix)	MAPE_P_ (%)	R_P_	RMSE_P_ (°Brix)	MAPE_P_ (%)	R_P_	RMSE_P_ (°Brix)	MAPE_P_ (%)
PLSR	0.5872	0.8378	6.29	0.5739	0.8400	5.91	0.6201	0.8031	5.55
SVR	0.5940	0.8407	6.09	0.5619	0.8468	5.83	0.6398	0.7934	5.56
GPR	0.6819	0.7637	5.47	0.7003	0.7650	5.52	0.6990	0.7427	5.29
1D-CNN	0.5139	1.4941	11.34	0.3847	1.1346	7.98	0.3894	1.3792	9.98

**Table 2:** Effect of feature-level fusion of spectral and color features on Hongmeiren SSC prediction.

Input	Model	Spectral Feature			Spectral Feature + Color Feature		
		R_P_	RMSE_P_ (°Brix)	MAPE_P_ (%)	R_P_	RMSE_P_ (°Brix)	MAPE_P_ (%)
Band1	GPR	0.6819	0.7637	5.47	0.6633	0.7770	5.55
Band2	GPR	0.7003	0.7650	5.52	0.7009	0.7487	5.58
Band3	GPR	0.6990	0.7427	5.29	0.7247	0.7173	5.18
All Bands	GPR	0.7444	0.6904	4.78	0.7414	0.7167	5.25
	CR-VGAFNet *	/	/	/	0.7744	0.6530	4.83

Note: / indicates not applicable; * indicates models that used OOF predictions from the base GPR models to estimate fusion parameters or train a second-level fusion model.

**Table 3:** Performance comparison of the decision-level fusion strategies and CR-VGAFNet.

Model	Spectral Feature			Spectral Feature + Color Feature		
	R_P_	RMSE_P_ (°Brix)	MAPE_P_ (%)	R_P_	RMSE_P_ (°Brix)	MAPE_P_ (%)
Average *	0.7578	0.7235	5.24	0.7377	0.7210	5.23
RMSE-based weighting *	0.7578	0.7233	5.24	0.7380	0.7205	5.23
Variance-based weighting *	0.7648	0.7166	5.22	0.7390	0.7191	5.23
Linear stacking (predictions) *	0.7496	0.6974	5.07	0.7323	0.7048	5.05
Linear stacking (predictions + variances) *	0.7367	0.7119	5.12	0.7227	0.7153	5.11
CR-VGAFNet *	/	/	/	0.7744	0.6530	4.83

Note: **/** indicates not applicable; * indicates models that used OOF predictions from the base GPR models to estimate fusion parameters or train a second-level fusion model.

**Table 4:** Ablation results involving the three functional components of CR-VGAFNet.

Model Number	Fusion Strategy	Color Residual	Variance Gate	Variance Attention	R_P_	RMSE_P_ (°Brix)	MAPE_P_ (%)
1	RMSE-based weighting *	×	×	×	0.7380	0.7205	5.23
2	RMSE-based weighting + CR *	√	×	×	0.7615	0.6705	4.79
3	VGAF *	×	√	√	0.7579	0.6826	4.89
4	AF (no variance gate) + CR *	√	×	√	0.7576	0.6746	4.83
5	VG + RMSE-based weighting + CR *	√	√	×	0.7574	0.6872	5.00
6	VGAF + CR *	√	√	√	0.7744	0.6530	4.83

Note: * indicates models that used OOF predictions from the base GPR models to estimate fusion parameters or train a second-level fusion model.

## Data Availability

The spectra, SSC values, model settings are available from the corresponding author upon reasonable request. The source code is not publicly available owing to ongoing instrument development; implementation details are provided in the manuscript and Supplementary Materials.

## Supplementary Materials

The following supporting information can be downloaded at: https://www.mdpi.com/article/10.3390/foods15173166/s1, Figure S1: Relationship between GPR predictive variance and prediction error for the independent prediction set; Table S1: Sample sources, dataset allocation, and use during model development and evaluation; Table S2: Optimized parameters of the models reported in manuscript; Table S3: Complete fold-specific GPR configurations used to generate OOF inputs; Table S4: Performance of different single-band Vis-NIR models for Hongmeiren SSC; Table S5: Performance of feature-level fusion of spectral and color features; Table S6: Performance comparison of decision-level fusion strategies and CR-VGAFNet; Table S7: Ablation results of VGAF and CR modules in CR-VGAFNet; Table S8: Comparison of predictive performance, model complexity, and inference efficiency of different attention-fusion models on the independent prediction set; Table S9: Prediction results of CR-VGAFNet for representative samples from a second orchard in Xiaotang Township.
