# Supplementary material for: Tri-Band Vis–NIR Spectroscopy with Color Residual-Variance Gated Attention Fusion for Rapid Assessment of Hongmeiren (Citrus reticulata) Soluble Solids Content
Source: Foods. 2026 Sep 7;15(17):3166. doi: 10.3390/foods15173166 (PMC13565095; doi:10.3390/foods15173166)
Supplement: Supplementary file 1 [file foods-15-03166-s001.zip › foods-4441674-supplementary.pdf]

Supplementary materials

Table S1. Sample sources, dataset allocation, and use during model development and evaluation.

Sample group	Source	Number of samples	Dataset allocation	Use in the study	Involved in model development
Primary dataset	Seven production areas	360	Calibration set and independent prediction set	Model development and primary performance evaluation	Yes
Calibration set	Selected from the primary dataset	268	Calibration samples	Preprocessing and hyperparameter selection, OOF feature generation, models training	Yes
Independent prediction set	Selected from the primary dataset	92	Independent prediction samples	Final performance evaluation only	No
Additional independent-site samples	Independent eighth production site	6	Separate supplementary dataset	Supplementary independent-site evaluation only	No
Total collected samples	Eight production sites	366	—	Primary and supplementary evaluations	—

Note: The six additional samples were not included in the original 360-sample dataset. They were collected from a different orchard in Xiaotang Township from the orchard that contributed the 14 Xiaotang Township samples to the primary dataset. They were not used for preprocessing selection, PCA fitting, hyperparameter optimization, OOF generation, neural-network training, early stopping, or model selection. All samples were transported to the laboratory within 48 h after harvest. For each fruit, spectral acquisition was followed immediately by SSC measurement.

Table S2. Optimized parameters of the models reported in manuscript.

Model	Input	Preprocessing	PCA components	Optimized parameters
PLSR	Band 1	1st derivative	15	Number of latent variables = 15
PLSR	Band 2	SG	8	Number of latent variables = 8
PLSR	Band 3	SG	12	Number of latent variables = 10
SVR	Band 1	Raw	12	RBF kernel; C = 0.599484; γ = 0.1; ε = 0.1
SVR	Band 2	SG	8	Linear kernel; C = 0.599484; ε = 0.01
SVR	Band 3	Raw	15	RBF kernel; C = 1.668101; γ = 0.1; ε = 0.1
GPR	Band 1	SNV	15	Matern(length scale = 10, ν = 2.5) + WhiteKernel(noise level = 0.5)
GPR	Band 2	1st derivative	10	Matern(length scale = 1, ν = 1.5) + WhiteKernel(noise level = 0.1)
GPR	Band 3	Raw	15	Matern(length scale = 1, ν = 1.5) + WhiteKernel(noise level = 0.1)
GPR	Band 1 + Color Features	SNV	15 + 6 Color Features	Matern(length scale = 10, ν = 2.5) + WhiteKernel(noise level = 0.5)
GPR	Band 2 + Color Features	1st derivative	10 + 6 Color Features	Matern(length scale = 1, ν = 1.5) + WhiteKernel(noise level = 0.1)
GPR	Band 3 + Color Features	Raw	15 + 6 Color Features	Matern(length scale = 1, ν = 1.5) + WhiteKernel(noise level = 0.1)
GPR	All Bands	Raw	40	RBF(length scale = 10) + WhiteKernel(noise level = 0.5)
GPR	All Bands + Color Features	Raw	40 + 6 Color Features	RBF(length scale = 10) + WhiteKernel(noise level = 0.5)
1D-CNN	Band 1	SG + 1st derivative	/	1 convolutional layer; 16 filters; kernel size = 3; FC units = 16; Dropout = 0.5
1D-CNN	Band 2	MSC	/	1 convolutional layer; 8 filters; kernel size = 3; FC units = 16; Dropout = 0.5
1D-CNN	Band 3	SNV	/	1 convolutional layer; 16 filters; kernel size = 3; FC units = 16; Dropout = 0.5

Table S3. Complete fold-specific GPR configurations used to generate OOF inputs.

Fold	Band	Preprocessing / PCA	Optimized parameters
1	1	Raw / 15	Matern(length scale = 4.1, ν = 1.5) + WhiteKernel(noise level = 0.3)
1	2	SG+MSC / 12	Matern(length scale = 4.9, ν = 1.5) + WhiteKernel(noise level = 0.4)
1	3	MSC / 12	RBF(length scale = 3.3) + WhiteKernel(noise level = 0.4)
2	1	Raw / 10	RBF(length scale = 4.8) + WhiteKernel(noise level = 0.5)
2	2	SNV / 12	RBF(length scale = 5.4) + WhiteKernel(noise level = 0.5)
2	3	SG / 10	Matern(length scale = 3.4, ν = 1.5) + WhiteKernel(noise level = 0.321)
3	1	Raw / 15	RBF(length scale = 4.4) + WhiteKernel(noise level = 0.3)
3	2	SNV / 8	Matern(length scale = 3.7, ν = 1.5) + WhiteKernel(noise level = 0.4)
3	3	SG / 12	Matern(length scale = 3.4, ν = 1.5) + WhiteKernel(noise level = 0.2)
4	1	MSC / 15	Matern(length scale = 4.4, ν = 2.5) + WhiteKernel(noise level = 0.4)
4	2	SG+SNV / 12	Matern(length scale = 4.6, ν = 1.5) + WhiteKernel(noise level = 0.4)
4	3	MSC / 12	Matern(length scale = 4.0, ν = 1.5) + WhiteKernel(noise level = 0.3)
5	1	SNV / 15	RBF(length scale = 3.9) + WhiteKernel(noise level = 0.4)
5	2	MSC / 5	RBF(length scale = 3.1) + WhiteKernel(noise level = 0.6)
5	3	SG+MSC / 12	RBF(length scale = 3.5) + WhiteKernel(noise level = 0.4)

Table S4. Performance of different single-band Vis-NIR models for Hongmeiren SSC.

Model	Band1 (< 950 nm)			Band2 (950 - 1637 nm)			Band3 (> 1637 nm)		
	R_{CV}	$RMSE_{CV}$ (°Brix)	$MAPE_{CV}$ (%)	R_{CV}	$RMSE_{CV}$ (°Brix)	$MAPE_{CV}$ (%)	R_{CV}	$RMSE_{CV}$ (°Brix)	$MAPE_{CV}$ (%)
PLSR	0.5678 ± 0.0688	0.7956 ± 0.0938	5.67 ± 0.79	0.5824 ± 0.0190	0.7879 ± 0.0500	5.55 ± 0.48	0.5773 ± 0.0363	0.7929 ± 0.0634	5.57 ± 0.53
SVR	0.5538 ± 0.1127	0.8062 ± 0.1068	5.66 ± 0.75	0.5758 ± 0.0230	0.7928 ± 0.0503	5.51 ± 0.50	0.5922 ± 0.1134	0.7752 ± 0.0933	5.26 ± 0.67
GPR	0.5928 ± 0.0746	0.7755 ± 0.0874	5.65 ± 0.56	0.5775 ± 0.0770	0.7900 ± 0.0864	5.75 ± 0.53	0.6178 ± 0.1052	0.7561 ± 0.0936	5.48 ± 0.52
1D-CNN	0.1863 ± 0.2201	1.4356 ± 0.3184	10.00 ± 2.86	0.2232 ± 0.1816	1.9324 ± 0.6696	13.80 ± 5.60	0.3415 ± 0.1206	1.3470 ± 0.1172	9.31 ± 1.01

Table S5. Performance of feature-level fusion of spectral and color features

Input	Model	Spectral Feature			Spectral Feature + Color Feature		
		R_{CV}	$RMSE_{CV}$ (°Brix)	$MAPE_{CV}$ (%)	R_{CV}	$RMSE_{CV}$ (°Brix)	$MAPE_{CV}$ (%)
Band1	GPR	0.5928 ± 0.0746	0.7755 ± 0.0874	5.65 ± 0.56	0.5929 ± 0.0720	0.7778 ± 0.0843	5.66 ± 0.60
Band2	GPR	0.5775 ± 0.0770	0.7900 ± 0.0864	5.75 ± 0.53	0.6105 ± 0.0489	0.7683 ± 0.0678	5.37 ± 0.49
Band3	GPR	0.6178 ± 0.1052	0.7561 ± 0.0936	5.48 ± 0.52	0.6337 ± 0.0865	0.7487 ± 0.0834	5.27 ± 0.51
All Bands	GPR	0.6789 ± 0.0732	0.7109 ± 0.0911	4.98 ± 0.54	0.6577 ± 0.0598	0.7371 ± 0.0776	5.18 ± 0.46

Table S6 Performance comparison of decision-level fusion strategies and CR-VGAFNet.

Model	Spectral Feature			Spectral Feature + Color Feature		
	R_{CV}	$RMSE_{CV}$ (°Brix)	$MAPE_{CV}$ (%)	R_{CV}	$RMSE_{CV}$ (°Brix)	$MAPE_{CV}$ (%)
Average	0.6288 ± 0.0837	0.7591 ± 0.0984	5.38 ± 0.47	0.6391 ± 0.0645	0.7485 ± 0.0912	5.27 ± 0.47
RMSE based weighting	0.6292 ± 0.0834	0.7588 ± 0.0984	5.38 ± 0.47	0.6396 ± 0.0650	0.7482 ± 0.0915	5.27 ± 0.47
Inverse variance based weighting	0.6347 ± 0.0761	0.7547 ± 0.0957	5.35 ± 0.47	0.6433 ± 0.0653	0.7453 ± 0.0904	5.25 ± 0.46
Linear Stacking (predictions)	0.6353 ± 0.0816	0.7491 ± 0.0926	5.33 ± 0.39	0.6334 ± 0.0836	0.7489 ± 0.0967	5.32 ± 0.49
Linear Stacking (predictions + variances)	0.6286 ± 0.0915	0.7532 ± 0.1016	5.29 ± 0.44	0.6318 ± 0.0904	0.7489 ± 0.0998	5.29 ± 0.50
CR-VGAFNet	/	/	/	0.6860 ± 0.0965	0.7067 ± 0.0894	5.10 ± 0.50

Table S7. Ablation results of VGAF and CR modules in CR-VGAFNet.

Model Number	Fusion Strategy	R_{CV}	$RMSE_{CV}$ (°Brix)	$MAPE_{CV}$ (%)
1	RMSE based weighting	0.6396 ± 0.0650	0.7482 ± 0.0915	5.27 ± 0.47
2	RMSE based weighting + CR	0.6508 ± 0.0868	0.7624 ± 0.0796	5.43 ± 0.44
3	VGAF	0.6457 ± 0.0817	0.7774 ± 0.0687	5.51 ± 0.36
4	AF (no gate) + CR	0.6344 ± 0.0980	0.7780 ± 0.0904	5.50 ± 0.50
5	VG + RMSE based weighting + CR	0.6710 ± 0.0818	0.7131 ± 0.0989	5.18 ± 0.49
6	VGAF + CR	0.6860 ± 0.0965	0.7067 ± 0.0894	5.10 ± 0.50

Table S8. Comparison of predictive performance, model complexity, and inference efficiency of different attention-fusion models on the independent prediction set.

Model	Trainable parameters	R_P	$RMSE_P$ (°Brix)	$MAPE_P$ (%)	Prediction time for 92 samples (ms)
CR-VGAFNet	32	0.7744	0.6530	4.83	0.2988
CBAM-style attention + CR	31	0.7571	0.6768	4.84	0.3640
MHA fusion + CR	104	0.7614	0.6705	4.79	0.3343

Note: All models were evaluated using the same OOF-generated GPR inputs, independent prediction set containing 92 samples, and fixed random seed of 42. Prediction time represents the median GPU inference time for the complete 92-sample batch after 200 warm-up runs and 1000 repeated measurements; file input/output and one-time model loading were excluded. Experiments were conducted under Windows 10 Pro using an Intel Core i5-13490F CPU, 16 GB RAM, and an NVIDIA GeForce RTX 3060 GPU with 12 GB memory. The software environment consisted of Python 3.11.5, PyTorch 2.6.0, CUDA 12.6, cuDNN 9.5.0, scikit-learn 1.7.1, and NumPy 2.0.1.

Table S9. Prediction results of CR-VGAFNet for representative samples from a second orchard in Xiaotang Township.

Sample ID	Predicted SSC (°Brix)	Measured SSC (°Brix)	Absolute prediction error (°Brix)	RMSE (°Brix)	MAPE (%)
A-L	9.965	10.6	0.635	-	-
A-M	9.996	10.2	0.204	-	-
A-S	10.045	10.5	0.455	-	-
B-L	10.038	10.2	0.162	-	-
B-M	10.010	9.7	0.310	-	-
B-S	11.126	12.9	1.774	-	-
Overall (n = 6)	-	-	-	0.808	5.14

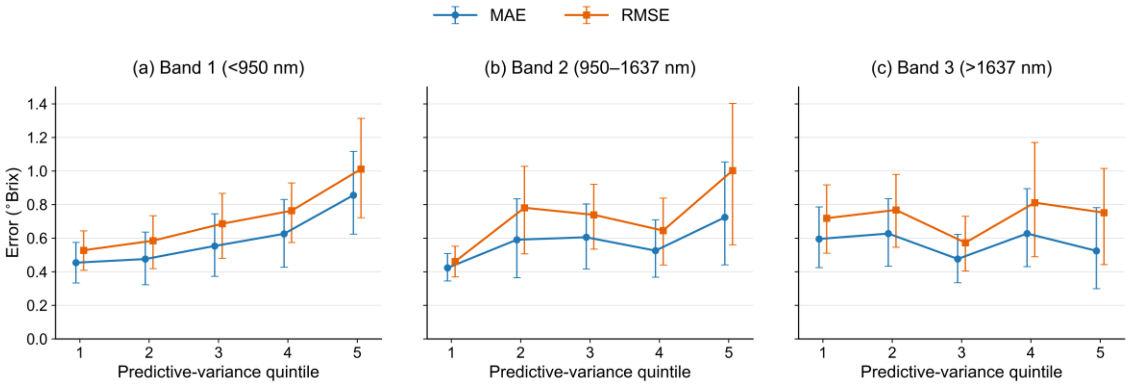

Figure S1. Relationship between GPR predictive variance and prediction error for the independent prediction set.
